# Expression of SREBP2 and cholesterol metabolism related genes in TCGA glioma cohorts

**DOI:** 10.1097/MD.0000000000018815

**Published:** 2020-03-20

**Authors:** Dali Li, Shenglan Li, Allen Z. Xue, Laura A. Smith Callahan, Ying Liu

**Affiliations:** a The Vivian L. Smith Department of Neurosurgery, McGovern Medical School; b Center for Stem Cell and Regenerative Medicine, the Brown Foundation Institute of Molecular Medicine, The University of Texas Health Science Center at Houston, Houston, Texas.

**Keywords:** diffuse glioma, GBM, SREBP, TCGA

## Abstract

Diffuse gliomas are the most common primary brain tumors. The Cancer Genome Atlas (TCGA) database provides correlative evidence between altered molecular pathways and gliomas. Dysregulated cholesterol homeostasis emerges as a potential indicator of the pathogenesis of gliomas.

Mining large cohorts from the TCGA together with database from the Chinese Glioma Genome Atlas (CGGA) for confirmation, we compared gene expression of cholesterol synthesis master regulator SREBP2 and its regulatory networks in low grade glioma (LGG) and glioblastoma (GBM).

Our analysis shows that expression of SREBP2 and related genes is lower in GBM than in LGG, indicating that cholesterol metabolism processes, including de novo synthesis, cholesterol uptakes, and cholesterol conversion and efflux, are suppressed in GBM.

Overall, our data suggests that SREBP2 transcript could serve as a potential prognosis marker or therapeutic target in diffuse glioma including GBM.

## Abstract 2

Supplemental Digital Content is available in the text

## 1 Introduction

Diffuse gliomas consist of low grade glioma (LGG, World Health Organization (WHO) histological grades II and III, i.e., G2 and G3) and glioblastoma (GBM, grade IV, or G4). LGG patients have a median survival of 7 years while GBM patients have a drastically reduced median survival of 14.6 months.^[1–3]^ In some cases, LGG progresses to secondary GBM and subsequently has a much worse prognosis than the primary LGG. Highly chemotherapy- and radiotherapy-resistant, diffuse gliomas remain among the deadliest human malignancies, despite significant strides over the last few decades in modern medicine. Comprehensive understandings at the molecular level of gliomas will facilitate the development of novel therapeutic targets in a more precise manner.

Large-scale genomic studies based on The Cancer Genome Atlas (TCGA) have allowed molecular mechanism research on large cohorts of cancer patients. Through profiling and analyzing large numbers of human tumors, aberrations at multiple levels (gene, transcription, protein, and epigenetic level) can be identified and applied to development of novel treatments.^[4–6]^ Recently, the WHO has introduced molecular parameters, such as mutations in the isocitrate dehydrogenase (IDH) 1 and 2 genes, and 1p/19q co-deletion, to define glioma entities.^[7]^ These updates reflect the consideration of genetic and genomic contributions to biological behaviors of gliomas.

Dysregulated lipid metabolism is a hallmark of malignant cancer.^[8–10]^ Cancer cells require a large amount of lipids for energy consumption and new membrane synthesis formation of daughter cells during division.^[11]^ Sterol response element-binding proteins (SREBPs) family of transcription factors are master regulators of endogenous synthesis of several major lipid categories including fatty acids, triglycerides, and cholesterol.^[12–20]^ In addition, SREBPs have been linked to the epidermal growth factor receptor (EGFR) mutations (EGFRvIII) and phosphoinositide 3-kinase (PI3K) hyperactivation, which have been shown to promote GBM tumor growth.^[21]^

SREBPs are basic-helix-loop-helix leucine zipper (bHLH-Zip) transcription factors that orchestrate lipid metabolism. Of the 3 isoforms of SREBPs, SREBP1a is a potent activator that activates both fatty acid and cholesterol synthesis, SREBP1c is specific for fatty acid synthesis, and SREBP2 mainly promotes cholesterol synthesis and uptake.^[12,22]^ In mammals, *SREBF1* encodes for both SREBP1a and SREBP1c, and *SREBF2* encodes for SREBP2.^[12,23]^ All SREBPs are first synthesized to be endoplasmic reticulum membrane (ER)-bound protein precursors, which subsequently undergo proteolytic cleavages in the Golgi apparatus, then function in the nucleus. The cleavage-activation process has not been completely understood however, SREBP2 has been shown that upon the stimulation of shortage of sterols, the precursor SREBP, originally in a complex with SREBP cleavage-activating protein (SCAP) in the ER membrane, is escorted by Insulin-induced gene proteins (INSIGs), to the Golgi apparatus and then activated by 2 proteolytic enzymes, site-1-protease (S1P) and site-2-protease (S2P). Therefore, maturation or activation of SREBPs can be controlled by the cellular sterol content. These reports collectively indicate that SREBP2 is regulated in a complex manner at multiple levels.^[12,22]^

SREBP1 has been shown to be a critical link between lipid metabolism and oncogenesis.^[13,14,21,24]^ However, the effects and potential relationship of *SREBP2*gene expression and cholesterol metabolism on overall survival, prognosis, or degree of malignancy have not been well characterized in diffuse glioma. Using a large cohort from TCGA together with a cohort from Chinese Glioma Genome Atlas (CGGA) for confirmation, we compared expression of SREBP2 and genes in the cholesterol regulatory networks in LGG vs GBM. Our analysis showed that SREBP2 mRNA expression was associated with mean survival of diffuse glioma patients, and that expression of genes involved in SREBP2-orchestrated cholesterol metabolism processes, including de novo synthesis, uptakes, conversion, and efflux, was all suppressed in GBM cells from the database samples.

## 2 Materials and methods

### 2.1 Datasets

All TCGA datasets were downloaded from UCSC Xena (http://xena.ucsc.edu/). The datasets included RNAseq gene expression, copy number variation (CNV), reverse phase protein array (RPPA) data from TCGA lower grade glioma, and glioblastoma (LGG and GBM, respectively) cohort. The transcription expression value from RNAseq (polyA + IlluminaHiSeq) was shown as log2 (x+1) transformed RSEM normalized count. RPPA data was normalized from the MDACC RPPA core. The gene-level CNV was estimated using the GISTIC2 method.^[25]^ Detailed data processing methods were described at the UCSC Xena website. An RNA-seq cohort from the Chinese Glioma Genome Atlas (CGGA) was downloaded from http://www.cgga.org.cn/.

### 2.2 Data analysis

Differential expression analysis among each group was performed using package limma of R.^[26,27]^ The complete R code is detailed in Supplementary data.

Heatmap and clustering were generated using package pheatmap. Functional enrichment analysis of differentially expressed genes (DEGs) was performed using The Database for Annotation, Visualization and Integrated Discovery (DAVID) to identify GO categories.^[28]^ GO terms were visualized by Revigo.^[29]^ False discovery rate (FDR) <0.05 was used as the cut-off. The protein–protein interaction (PPI) network was retrieved from STRING database and reconstructed in Cytoscape software.^[30,31]^ Although graphs of PPI network could be derived directly from STRING database, to better visualize protein–protein interactions, Cytoscape software was used to reconstruct the Figures presented in this manuscript. Ethical approval for the study of using online databases was granted from institutional review board at UTHealth-Houston.

### 2.3 Statistics

Results were compared between the groups using Student *t* test. Overall survival was analyzed using the Kaplan–Meier method. Statistical analysis was performed with the Graphpad software.

## 3 Results

### 3.1 High expression of SREBP2 predicted favorable prognosis in diffuse gliomas

**Figure 1:**
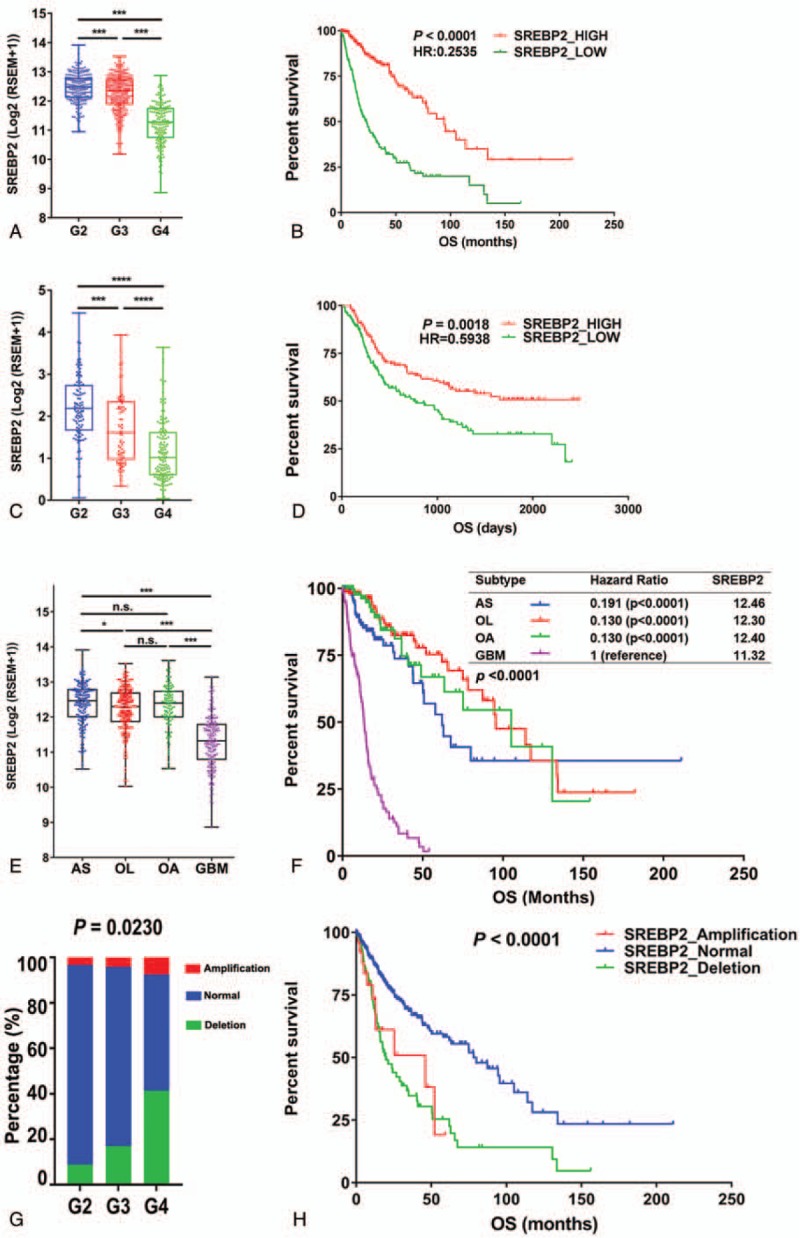
Expression of SREBP2 is associated with overall survival of diffuse glioma cases in both TCGA and CGGA datasets. (A) mRNA expression of SREBP2 in gliomas of WHO grade G2, G3, and G4 (GBM) in TCGA dataset. Grade 2, n = 216; Grade 3, n = 241; Grade 4, n = 160, Student *t* test. (B) Kaplan–Meier survival curves of SREBP2 high/low expression groups in diffuse glioma patients from the TCGA dataset. (C) mRNA expression of SREBP2 in gliomas of WHO grade G2, G3, and G4 (GBM) in the CGGA dataset. Grade 2, n = 105; Grade 3, n = 67; Grade 4, n = 138, Student *t* test. (D) Kaplan–Meier survival curves of SREBP2 high/low expression groups in diffuse glioma patients from the CCGA dataset. (E) mRNA expression of SREBP2 in different glioma subtypes in the TCGA dataset. Astrocytoma (AS), n = 169, oligodendroglioma (OL), n = 174, oligoastrocytoma (OA), n = 114, GBM, n = 160. Student *t* test. The groups were divided according to the median level of SREBP2 mRNA expression. (F) Kaplan–Meier survival curves of different subtypes of glioma. Inset table shows hazard ratio (HR) of the comparison of each subtype with GBM, and the Log2 median expression level of SREBP2 (Table in panel F). (G) of SREBP2 in LGG and GBM. Percentage of Copy number variation (CNV) events of SREBP2 in LGGs and GBMs. SREBP2 amplification, n = 35; SREBP2 normal, n = 458; SREBP2 deletion, n = 124. (H) Kaplan–Meier survival curves of patients with different CNV events. HR was calculated by the ratio of overall survival (OS) of each subtype group vs GBM over the study time period. Ticks represent censored values. AS, astrocytoma, OL, oligodendroglioma, OA, oligoastrocytoma. ^∗^, *P* < .05; ^∗∗^, *P* < .01; ^∗∗∗^, *P* < .001; ^∗∗∗∗^, *P* < .0001; n.s., not significant.

Clinicopathological information of 457 LGG patients (216 cases of G2, 241 cases of G3) and 160 cases of GBMs from TCGA database is summarized in Table 1. To examine if there was a correlation of SREBP2 expression with diffuse glioma clinical grades, we compared SREBP2 mRNA level along glioma grades, G2, G3, and G4 (GBM). It is interesting to note that as glioma grades increased, the mRNA expression of SREBP2 was significantly decreased (*P* < .001 for G2 vs G3, G3 vs GBM, and G2 vs GBM) (Fig. 1A). More intriguingly, survival analysis showed that patients with higher SREBP2 expression had an increased overall survival (*P* < .0001, HR = 0.2535) (Fig. 1B). These results indicate that SREBP2 mRNA expression was negatively correlated with malignancy grading and that higher SREBP2 transcript level could predict favorable prognosis in diffuse glioma.

**Table 1:**
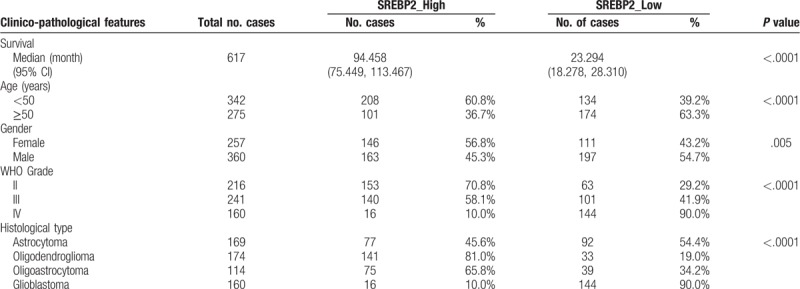
Clinical information of 617 diffuse glioma cases from TCGA database.

To confirm these observations, we obtained a second diffuse glioma dataset from the Chinese Glioma Genome Atlas (CGGA). The CGGA glioma cohort (Table 2) consisted of 172 LGG cases and 138 cases of GBM patients, a majority of whom were of Eastern or Asian ethnicities. Consistent with the results from TCGA database, the expression level of SREBP2 decreased from G2 to GBM in the CGGA cohort (Fig. 1C, *P* < .05). Encouragingly, high expression of SREBP2 transcript also predicted better prognosis in the CGGA dataset, again consistent with results obtained from TCGA glioma cohort (Fig. 1D).

**Table 2:**
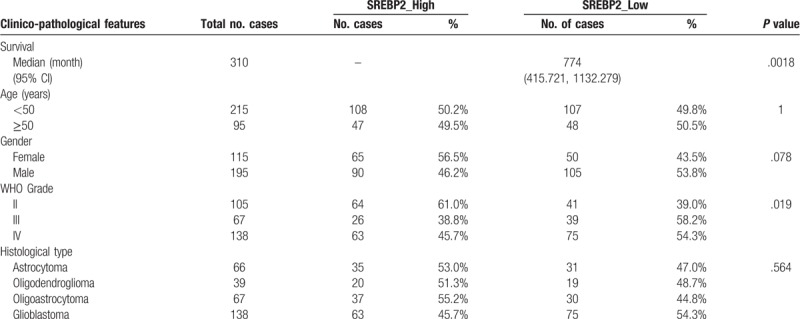
Clinical information of 310 diffuse glioma cases from CGGA database.

SREBP2 is shown to control cholesterol synthesis, which is differentially regulated in oligodendrocytes and astrocytes. To correlate mRNA expression of SREBP2 in different histological types across diffuse gliomas, we plotted gene expression of SREBP2 with astrocytoma, oligodendrogliomas, oligoastrocytomas, and GBM (Fig. 1E). Our results showed that SREBP2 was expressed at significantly lower levels in oligodendrogliomas or oligoastrocytomas than in astrocytomas (Fig. 1E, Student *t* test, *P* < .05). Furthermore, all 3 histological types of low grade gliomas had much higher SREBP2 expression than GBMs (*P* < .05 or *P* < .001, Fig. 1E). Taken together, our analysis shows that SREBP2 was expressed at a significantly higher level in LGGs than in GBMs, regardless of histological subtypes.

To correlate overall mean survival of histological LGG subtypes and GBMs with SREBP2 expression, we plotted Kaplan–Meier survival curves for astrocytoma, oligodendrogliomas, oligoastrocytomas, and GBM, respectively (Fig. 1F). Because astrocytoma is reportedly most similar to GBM in terms of histology, we first compared astrocytoma with GBM for overall survival. SREBP2 was expressed at higher levels in astrocytoma than in GBM (Fig. 1E, F, *P* < .001), meanwhile, astrocytoma had a significantly better overall survival than GBM (Fig. 1F, hazard ratio = 0.191, *P* < .0001). Additional comparison showed that that SREBP2 is a predictor of favorable prognosis in diffuse glioma, regardless of histology subtypes.

### 3.2 Distinct copy number variations of SREBP2 in GBM and LGG

DNA copy number variations (CNVs) are an important component of alterations in gene expression.^[32]^ Therefore, we compared CNVs of SREBP2 in LGG and GBM. More deletion events of SREBP2 were found in GBM patients, and the rate of deletion events of SREBP2 reached more than 40% in GBM (Fig. 1G). Furthermore, the number of deletion events of SREBP2 negatively correlated with overall survival (Fig. 1H). Hence, our data indicated that, in addition to SREBP2 mRNA expression level, SREBP2 CNVs (both the nature and the frequency of events) also had the potential to be developed into prognosis markers in diffuse gliomas.

### 3.3 Genes related to de novo cholesterol synthesis were expressed at lower levels in GBM than in LGG

**Figure 2:**
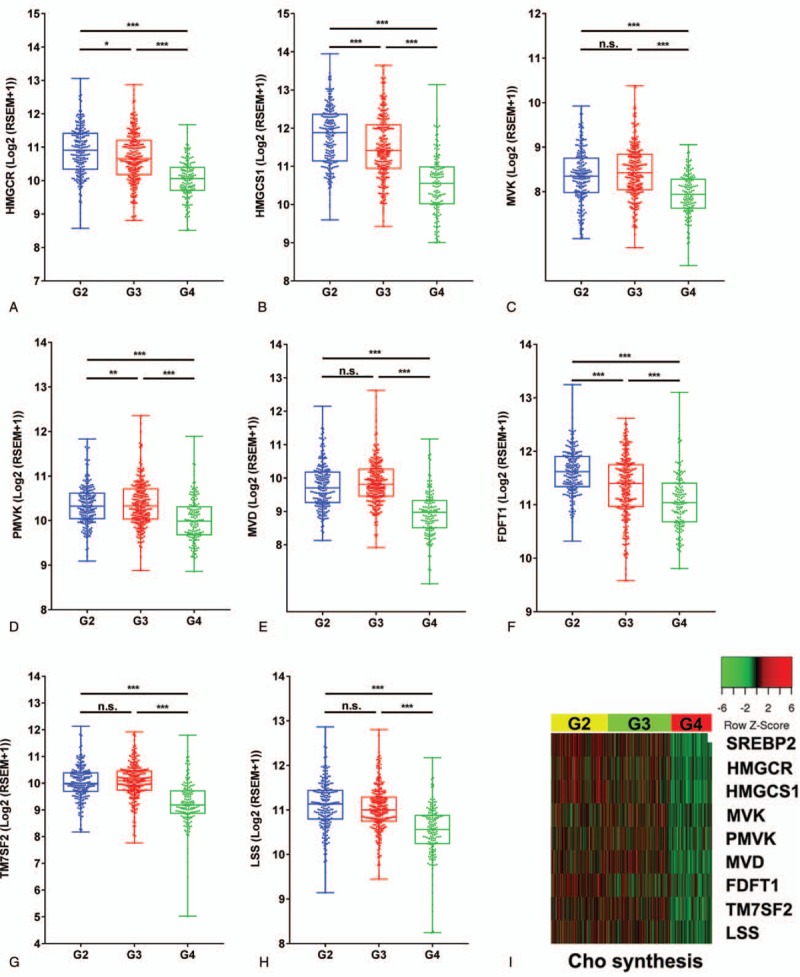
SREBP2 and genes of de novo cholesterol synthesis is expressed at lower levels in GBM than in LGG. (A-H) Box plots of mRNA expression of cholesterol synthesizing enzyme coding genes (HMGCR, HMGCS1, FDFT1, LSS, and CYP46A1) in LGG (G2 and G3) and GBM from TCGA database. (I) Heatmap shows that expression of SREBP2 and genes involved in cholesterol synthesis is significantly lower in GBM than in LGG. WHO Grade 2, n = 216; Grade 3, n = 241; Grade 4, n = 160, Student *t* test, ^∗^, *P* < .05; ^∗∗^, *P* < .01; ^∗∗∗^*P* < .001; n.s., not significant.

SREBP2 orchestrates the expression of multiple enzymes in cholesterol biosynthetic pathway.^[33,34]^ If gene expression of SREBP2 is low, then enzymes in the downstream lipid pathways could also be down-regulated. We compared the mRNA expression of these enzymes in LGG and GBM from TCGA cohort. As expected, gene expression of enzymes for cholesterol synthesis, including HMGCS1, HMGCR, MVK, PMVK, MVD, FDFT1, TM7SF2, and LSS, was accordingly decreased in GBM samples (Fig. 2). These together suggested that GBM had lower levels of de novo cholesterol synthesis than LGGs, at least at the mRNA transcription level.

### 3.4 mRNA expression of genes involved in cholesterol uptake was significantly lower in GBM than in LGG

**Figure 3:**
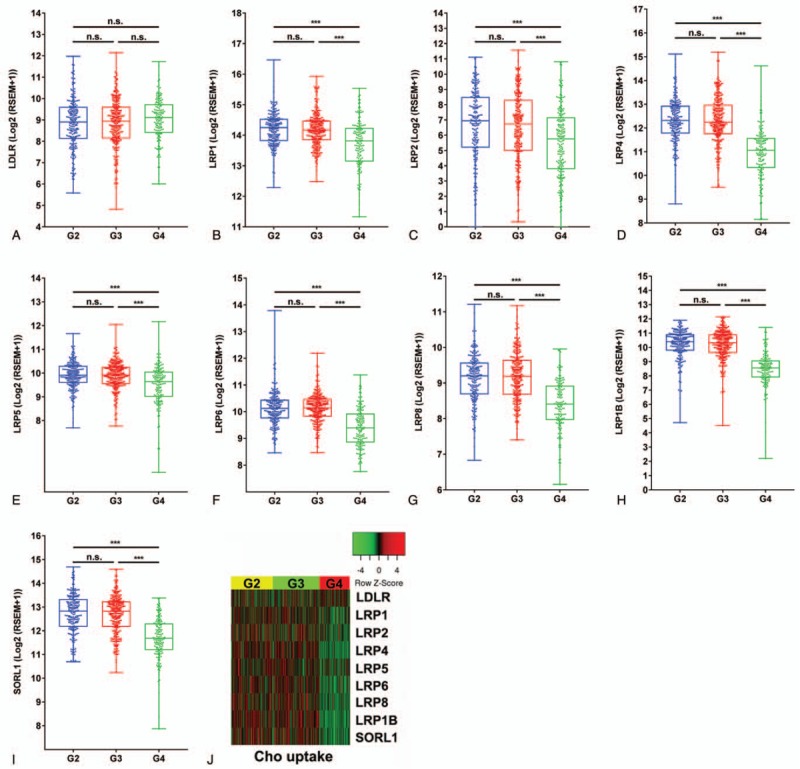
Cholesterol uptake in GBM is similar to that in LGG. (A-I) Box plots of mRNA expression of cholesterol transporters (LDLR, LRP1, LRP2, LRP4, LRP5, LRP6, LRP8, LRP1B, and SORL1) show that gene expression is similar in LGG and GBM from TCGA database. (J) Heatmap shows expression of cholesterol uptake is not significantly different in LGG and GBM. WHO Grade 2, n = 216; Grade 3, n = 241; Grade 4, n = 160, Student *t* test, ^∗^, *P* < .05; ^∗∗^, *P* < .01; ^∗∗∗^*P* < .001; n.s., not significant.

Besides de novo synthesis, uptake is also a major source for cells to obtain cholesterol. apolipoprotein E (ApoE) receptors family is reported to be key transporters for exogenous cholesterol uptake.^[21,35–37]^ Our data showed that LDLR mRNA expression was similar in GBM and LGG (Fig. 3). However, examination of gene expression of additional ApoE receptor family,^[36,38]^ such as LRP1, LRP2 (megalin), LRP4, LRP5/LRP6, apoER2/LRP8, LRP1B, and SORL1, showed that they were all down-regulated in GBM compared with LGG (Fig. 3).

### 3.5 Expression of genes involved in cholesterol excretion was lower in GBM than in LGG

**Figure 4:**
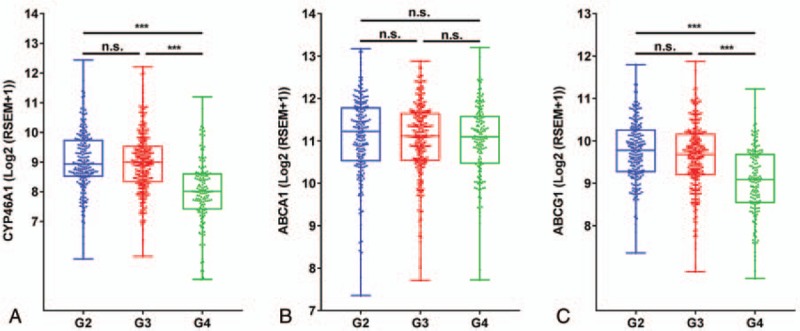
Expression of genes involved in cholesterol excretion is lower in GBM. Conversion to oxysterol is the major outlet for cholesterol excretion in the brain. CYP46A1 is the major enzyme in the brain that converts cholesterol to 24-hydroxycholesterol (24-OHC). CYP46A1 (A) and ABCG1 (C) mRNA is expressed higher in LGG than in GBM, while expression of ABCA1 (B), the other major gene involved in cholesterol excretion, is not significantly different between LGG and GBM. WHO Grade 2, n = 216; Grade 3, n = 241; Grade 4, n = 160, Student *t* test, ^∗^, *P* < .05; ^∗∗^, *P* < .01; ^∗∗∗^*P* < .001; n.s., not significant.

In the brain, when cholesterol acquisition exceeds functional needs, excretion occurs.^[39]^ Conversion to oxysterol is the major outlet for cholesterol excretion and CYP46A1 is the main enzyme that converts cholesterol to 24-hydroxycholesterol (24-OHC).^[40]^ Our data showed that CYP46A1 mRNA level was significantly lower in GBM than in LGG (Fig. 4). The other pathway associated with cholesterol turnover is secretion via ABC transporters, especially ABCA1 and ABCG1.^[40]^ ABCG1, but not ABCA1 was expressed at a lower level in GBM than in LGG (Fig. 4). These observations could potentially explain how GBM cells were able to meet their intracellular cholesterol needs with both depressed de novo synthesis (as a consequence of low expression of SREBP2) and depressed exogenous uptake. Thus, genes involved in cholesterol metabolism processes, including de novo synthesis, uptake, conversion, and efflux, were all expressed at a significantly lower level in GBM than in LGG.

### 3.6 Distinct genetic profiling in SREBP2-high and SREBP2-low expression gliomas

**Figure 5:**
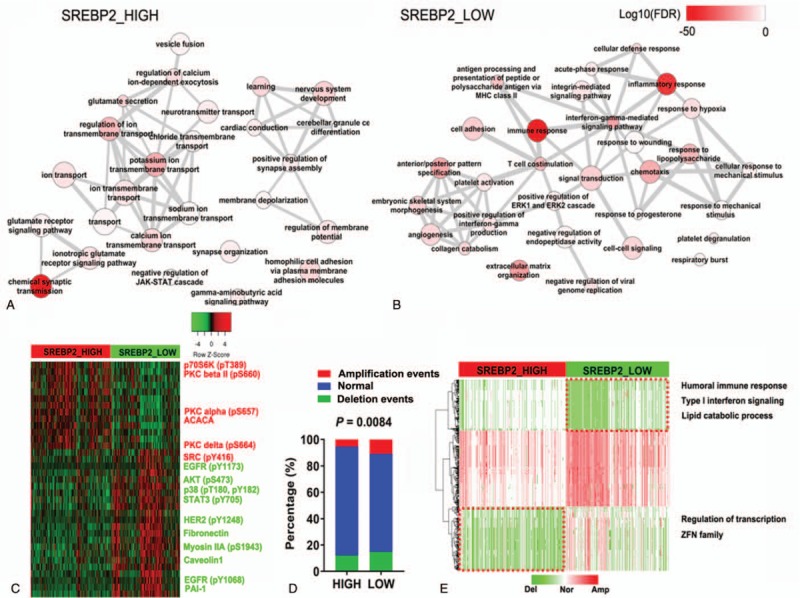
Distinct genetic profiling of SREBP2 high and low expression groups. (A, B) Biological process (BP) terms involved in SREBP2 high (a) or low (B) expression group. (C) Protein expression and phosphorylation (RPPA) in SREBP2 high or low expression groups n = 331. (D) Percentage of different CNV events in SREBP2 high or low expression groups. (E) Frequent amplification or deletion events in SREBP2 high or low expression groups.

Our results indicated that SREBP2 expression was distinct in GBM and LGG. To genetically profile SREBP2 in diffuse gliomas, we divided all gliomas of our TCGA cohort into 2 groups based on their SREBP2 gene expression level. The comparison between SREBP2-high vs SREBP2-low groups yielded 2427 differentially expressed genes (DEGs), of which 1157 genes were expressed at significantly higher, and 1270 genes were expressed at significantly lower levels, in SREBP2-high group. Functional enrichment analysis indicated that the SREBP2-high group had genetic signatures specific to neural lineages, and GO terms yielded from SREBP2-high group belonged to nervous system development and function (Fig. 5A, Supplementary Fig. S1). In addition, according to the reverse phase protein array (RPPA) data of the same TCGA cohort, SREBP2-high expression group had higher phosphorylation level of p70 S6K (pT389), PKCα (pS657), PKCβII (pS660), and PKCδ (pS664) (Fig. 5C), which is consistent with previous reports showing that SREBP2 high expression group had high expression of genes in the AMPK and PKC signaling pathways.^[41–44]^ In contrast, the SREBP2-low expression group had high expression of inflammation and extracellular matrix (Fig. 5B) molecules (e.g., PAI-1, caveolin1, and fibronectin), which was also confirmed by the RPPA data for expression at the protein level (Fig. 5C). In addition, phosphorylation of EGFR (pY1068 and pY1173), HER2 (pY1248), STAT3 (pY705), p38 (pT180 and pY182), AKT (pS473), Myosin IIA (pS1943), and SRC (pY416) was also highly enriched in SREBP2-low but not in SREBP2-high group (Fig. 5C). Increased copy number amplification and deletion events were observed in SREBP2-low expression group (Fig. 5D). Genes with deletion events were associated with humoral immune response, type I interferon signaling and lipid catabolic process (Fig. 5E, Supplementary Fig. S2) in SREBP2-low expression group, while more deletion events were found in zinc finger family genes (Fig. 5E, Supplementary Fig. S2) in SREBP2-high expression group, indicating potentially altered expression of additional transcription factors in this group. Taken together, these data suggested that SREBP2-high and low expressing diffuse glioma groups had distinct genetic profiles.

## 4 Discussion

The ability to access big database such as TCGA has empowered researchers to characterize molecular signature of tumor samples in a more precise manner. In the current work, we examined mRNA expression of SREBP2, the master transcription regulator that orchestrates cholesterol synthesis and transport, in large cohorts of GBM and LGG obtained from TCGA database. Our results showed that GBM and LGG had distinct mRNA expression profiles of SREBP2 and genes involved in cholesterol metabolism. Strikingly, SREBP2 and genes responsible for de novo cholesterol synthesis were all expressed at lower levels in GBM than in LGG. In addition, our analysis indicated that SREBP2 transcript level and CNVs significantly correlated with malignancy and overall survival of diffuse glioma.

A critical observation in our work in GBM is that genes involved in major metabolic aspects of cholesterol, including de novo synthesis and uptake, conversion to 24-OHC, and efflux via ABC transporter, were all expressed at very low levels. On the contrary, the mRNAs of these genes were expressed at relatively high levels in LGG. The underlying mechanisms of such a spectrum of changes on gene expression along diffuse glioma (G2, G3, GBM) for cholesterol metabolism from metabolically active to inert state could provide clues for pathogenesis of GBM malignancy or the progression of LGG to secondary GBM.

Our observation on the distinct gene expression profile of SREBP2 and genes in its in cholesterol metabolism network echoes with 2 previous reports, which found that cholesterol level was significantly lower in tumor tissues than their surrounding normal brain areas in patient samples.^[45,46]^ These reports suggest that glioma cells might have lower levels of cholesterol synthesis than normal brain cells. Since biosynthesis of cholesterol is an energy-expensive complex process in the cell,^[38]^ it is reasonable that glioma cells reduce their cholesterol synthesis to a minimally needed level. The conserved energy from depressed cholesterol synthesis could be used to make other essential components in cancer cell replication such as nucleic acids and proteins. Nygren et al showed up to 100-fold increase in the concentration of cholesterol ester, the transport form of cholesterol, in tumor-tissue and surrounding areas compared to control tissues.^[46]^ This could explain why mRNA expression of CYP46A1, the enzyme that coverts cholesterol to oxysterol, was also lower in GBM than in LGG in our study.

Based on our analysis and previous reports,^[45,46]^ a potential explanation could be that diffuse glioma cells may inherit the cholesterol metabolic feature from their original transformed neural cells and even reduce their requirement for cholesterol to a physiologically minimal level. The next step is to validate our gene expression analysis at the protein as well as posttranslational modification level in clinical patient samples in collaboration with neurosurgeons, which could include in-depth profiling of global phosphoproteomics^[47,48]^ of large cohorts of GBM tumor samples, together with matched LGG and normal controls.

## 5 Conclusion

In this study, expression of SREBP2 and genes involved in cholesterol synthesis process are significantly associated with prognosis in diffuse glioma cases obtained from TCGA database. Cholesterol metabolism, also, appears to be suppressed in GBMs. Analysis of additional independent databases, such as the CGGA, was used to confirm these results. Big data analyses using widely available TCGA and CGGA brain tumor databases reveal previously overlooked, yet unique characteristics of cholesterol metabolism in gliomas, which could provide new avenues for therapeutic intervention development and clinical prognosis.

## Author contributions

**Conceptualization:** Dali Li, Shenglan Li, Laura A Smith Callahan, Ying Liu.

**Data curation:** Dali Li, Shenglan Li.

**Formal analysis:** Dali Li, Shenglan Li.

**Funding acquisition:** Laura A Smith Callahan, Ying Liu.

**Investigation:** Dali Li, Shenglan Li, Allen Z Xue, Laura A Smith Callahan.

**Methodology:** Dali Li, Shenglan Li, Allen Z Xue, Laura A Smith Callahan.

**Project administration:** Laura A Smith Callahan, Ying Liu.

**Resources:** Dali Li, Shenglan Li.

**Software:** Dali Li, Shenglan Li.

**Supervision:** Laura A Smith Callahan, Ying Liu.

**Validation:** Dali Li, Shenglan Li.

**Visualization:** Dali Li, Shenglan Li.

**Writing – original draft:** Dali Li, Shenglan Li, Allen Z Xue, Laura A Smith Callahan, Ying Liu.

**Writing – review & editing:** Dali Li, Shenglan Li, Allen Z Xue, Laura A Smith Callahan, Ying Liu.

Ying Liu orcid: 0000-0003-1875-8293.

## Supplementary Material

Supplemental Digital Content

## Supplementary Material

Supplemental Digital Content

## Supplementary Material

Supplemental Digital Content
