# Supplementary material for: Expression of SREBP2 and cholesterol metabolism related genes in TCGA glioma cohorts
Source: Medicine (Baltimore). 2020 Mar 20;99(12):e18815. doi: 10.1097/MD.0000000000018815 (PMC7220679; doi:10.1097/MD.0000000000018815)
Supplement: Supplemental Digital Content [file medi-99-e18815-s001.docx]

**Supplementary data**

**Expression of SREBP2 and cholesterol metabolism related genes in TCGA glioma cohorts**

Dali Li, Shenglan Li, Allen Z. Xue, Laura A. Smith Callahan, and Ying Liu

**Supplementary methods:**

The complete R code is the following:

group <- read.csv("group.csv", header=T, row.names=1)

design=model.matrix(~0+factor(group$Disease))

colnames(design)=c('High','Low')

    fit=lmFit(sCLLex,design)

    cont.matrix=makeContrasts('High-Low',levels = design)

    fit2=contrasts.fit(fit,cont.matrix)

    fit2=eBayes(fit2)

    options(digits = 4)

    topTable(fit2,adjust='BH')
