# Supplementary material for: Expression of SREBP2 and cholesterol metabolism related genes in TCGA glioma cohorts
Source: Medicine (Baltimore). 2020 Mar 20;99(12):e18815. doi: 10.1097/MD.0000000000018815 (PMC7220679; doi:10.1097/MD.0000000000018815)
Supplement: Supplemental Digital Content [file medi-99-e18815-s002.doc]

**Supplementary Figure S1**. Cellular component (CC) and molecular function (MF) terms clustered from DEGs extracted from SREBP2 high (A, B) or low (C, D) expressing groups.

**
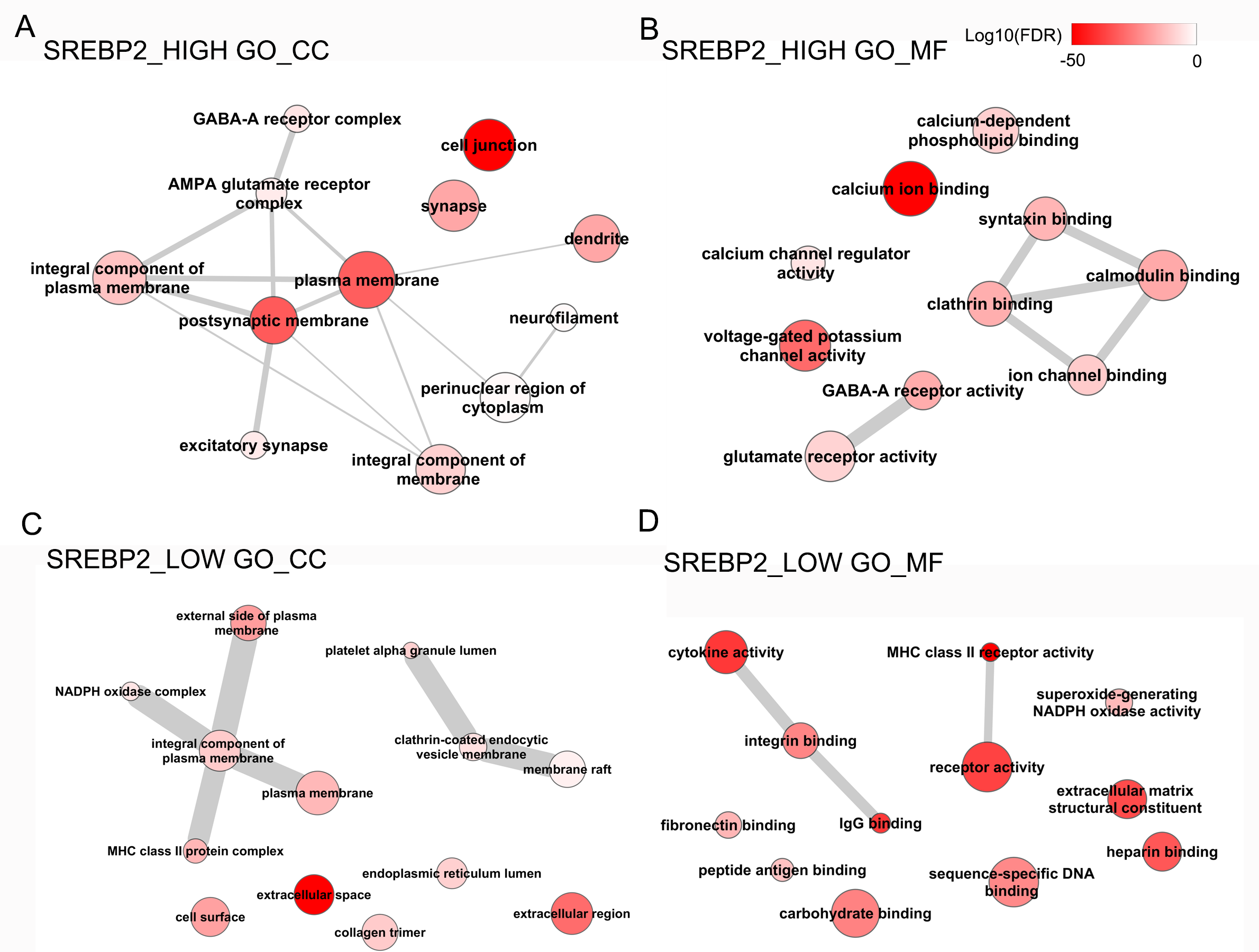
**
