# Supplementary material for: Expression of SREBP2 and cholesterol metabolism related genes in TCGA glioma cohorts
Source: Medicine (Baltimore). 2020 Mar 20;99(12):e18815. doi: 10.1097/MD.0000000000018815 (PMC7220679; doi:10.1097/MD.0000000000018815)

**Supplementary Figure S2**. CNV events of ZNF628 (A), ZNF524 (B), ZNF579 (C), EGFR (D), CDNK2A (E), CDNK2B (F), INFA1 (G), and INFA2 (H) in SREBP2 high or low expressing groups.


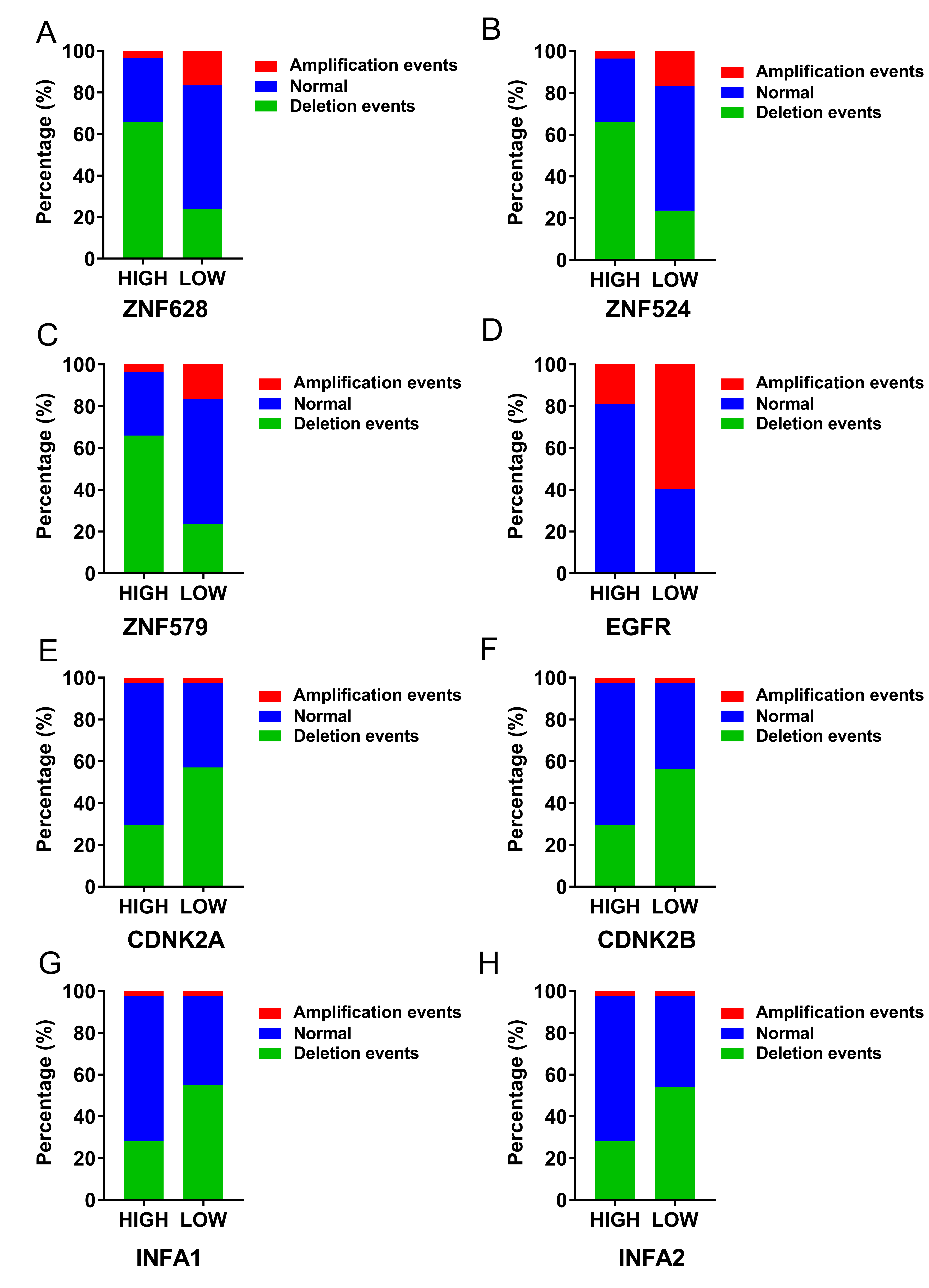

Supplement: Supplemental Digital Content [file medi-99-e18815-s003.doc]
